# Global prevalence of nocturnal enuresis and associated factors among children and adolescents: a systematic review and meta-analysis

**DOI:** 10.1186/s13034-025-00880-x

**Published:** 2025-03-20

**Authors:** Molalign Aligaz Adisu, Tesfaye Engdaw Habtie, Melesse Abiye Munie, Molla Azmeraw Bizuayehu, Alemu Birara Zemariam, Yabibal Asfaw Derso

**Affiliations:** 1 Department of Pediatrics and Child Health Nursing, College of Health Sciences, Woldia University, Woldia, Ethiopia; 2 Department of Nursing, College of Health Sciences, Woldia University, Woldia, Ethiopia

**Keywords:** Nocturnal enuresis, Global, Prevalence, Children, Adolescents, Meta-analysis

## Abstract

**Background:**

Nocturnal enuresis (NE), a prevalent childhood condition associated with significant emotional morbidity, including anxiety, guilt, and diminished self-esteem. Notably, NE exhibits substantial variability in prevalence across diverse geographical and sociocultural contexts ranging from 2 to 75%, highlighting the influence of environmental and societal factors. The associated social stigma exacerbates emotional distress, negatively impacting self-perception and overall quality of life. This systematic review and meta-analysis seek to synthesize global epidemiological data on NE, accounting for inter-country prevalence variations, and to elucidate its associated factors, thereby informing the development of culturally sensitive and effective intervention strategies.

**Methods:**

All observational quantitative research articles conducted among children and adolescents in the world were included. We used PubMed Central, Cochrane Library, Scopus, and Google Scholar searching databases. The study quality was checked using the Newcastle - Ottawa Scale. Then I^2^ statistics and Cochran’s Q test were used to evaluate heterogeneity. Funnel, Egger’s test, and non-parametric trim and fill effect tests were used to check publication bias by using a random effect model. Finally, subgroup analysis was done to evaluate statistical heterogeneity, and sensitivity analysis was also done to detect the presence or absence of any influential study.

**Results:**

In the final analysis, one hundred twenty-eight studies involving 445,242 individuals in 39 countries. The overall pooled prevalence of Nocturnal enuresis among children and adolescents was 7.2% (95% CI: 6.2-8.1%). Positive family history AOR 1.49 (95% CI: 1.26–1.71), positive urinary tract infection AOR; 3.89, 95% CI (2.93–4.46), parental death AOR = 1.93 (95% CI: 1.73–2.12), first birth order AOR 0.5 (95% CI: 0.37–0.62), and male sex AOR 1.63; 95% CI (1.31–1.94 were the significant associated factors with Nocturnal enuresis among children and adolescent.

**Conclusion:**

The study found that nocturnal enuresis affects approximately 7.2% of children and adolescents. Family history, urinary tract infection, parental death, birth order, and sex were statistically significant factors. It is recommended that healthcare providers should implement routine screening for nocturnal enuresis, particularly for children with known risk factors such as family history and urinary tract infections, and the development of targeted interventions and support mechanisms should be prioritized, considering the significant impacts of these factors among children and adolescents.

**Supplementary Information:**

The online version contains supplementary material available at 10.1186/s13034-025-00880-x.

## Introduction

Nocturnal enuresis (NE), or bedwetting, is defined by the DSM-5 as the involuntary loss of urine during sleep in children aged five and older; occurring at a developmental stage where bladder control is expected. Classified as an elimination disorder, NE is diagnosed when this behavior occurs at least twice a month and is notattributed to a medical condition or substance effects. NE is categorized into primary nocturnal enuresis, where children have never achieved consistent nighttime dryness, and secondary nocturnal enuresis, which occurs after a period of dryness lasting six months or more [1, 2].

Additionally, NE can be further classified based on associated symptoms; monosymptomatic nocturnal enuresis occurs without urological symptoms or daytime incontinence, while polysymptomatic nocturnal enuresis is linked to daytime bladder dysfunction such as urgency, frequency, or incontinence. Children with monosymptomatic NE may also experience psychosocial issues, including oppositional defiant disorder (ODD) or attention deficit hyperactivity disorder (ADHD). Understanding these classifications and underlying factors is essential for developing tailored therapeutic interventions for affected children [3, 4].

Globally, the prevalence of nocturnal enuresis varies considerably, influenced by factors such as age, gender, and cultural background. Research indicates that approximately 10–20% of children aged 5 years’ experience NE, with rates declining as children grow older. By adolescence, the prevalence drops to about 1–3% [5, 6]. Variations in prevalence rates can be attributed to differences in study designs, definitions of enuresis, and cultural perceptions surrounding the condition. Some studies suggest that boys are more likely to be affected than girls, with boys exhibiting higher rates of enuresis, particularly in younger age groups [6].

The consequences of nocturnal enuresis extend beyond the physical symptoms. Children who experience bedwetting may suffer from significant emotional distress, leading to low self-esteem and social withdrawal. Parents often report feelings of frustration and helplessness, which can strain family dynamics. The stigma associated with bedwetting can result in bullying and social exclusion, further exacerbating the psychological impact on affected children. As awareness of mental health issues grows, it is crucial to recognize the emotional and social ramifications of nocturnal enuresis and address them as part of a comprehensive treatment plan [7].

Understanding the factors that contribute to nocturnal enuresis is essential for effective management. Numerous studies have identified a range of associated factors, including genetic predispositions, developmental delays, vitamin D status, and psychological issues. Vitamin D receptors are present in detrusor muscle and urothelium of bladder. Vitamin D decrease detrusor contractions by suppressing sensory signals during filling phase. The vitamin D deficiency can increase uninhibited bladder contraction. A study defined that the vitamin D value was lower in Primary MNE children. It seems that vitamin D status was risk factor for development of PMNE [8]. Family history plays a significant role, with children having a higher risk of bedwetting if one or both parents experienced the condition during childhood [9, 10]. Psychological factors, such as stress, anxiety, and emotional disturbances, can also influence the onset and persistence of nocturnal enuresis [11]. Additionally, physiological factors, including bladder capacity and sleep disorders, have been implicated in the condition [12]. Another common and important disease in such children is functional Constipation which can be seen simultaneously with incontinency that it causes more anxiety in child and parents [13].

Cultural attitudes towards bedwetting can greatly influence how families perceive and manage NE. In some cultures, bedwetting is seen as a normal part of childhood, while in others, it may carry a significant stigma. This cultural context can affect the willingness of families to seek medical help and the types of interventions pursued. Treatment options for nocturnal enuresis vary widely, ranging from behavioral techniques and parental education to pharmacological interventions [14]. Recent advances in understanding the underlying mechanisms of enuresis have led to more effective treatment modalities, yet many children still go untreated.

Nocturnal enuresis is a hidden public health concern that warrants greater attention from healthcare professionals, educators, and society as a whole. The complex interplay of genetic, psychological, and physiological factors contributes to the prevalence of this condition among children and adolescents. By increasing awareness and understanding of nocturnal enuresis, we can improve the quality of life for affected children and their families [15]. This systematic review and meta-analysis aim to provide a comprehensive overview of the global prevalence of nocturnal enuresis and its associated factors, ultimately contributing to more effective management strategies and interventions.

## Methods

### Study design and reporting

**Fig. 1:**
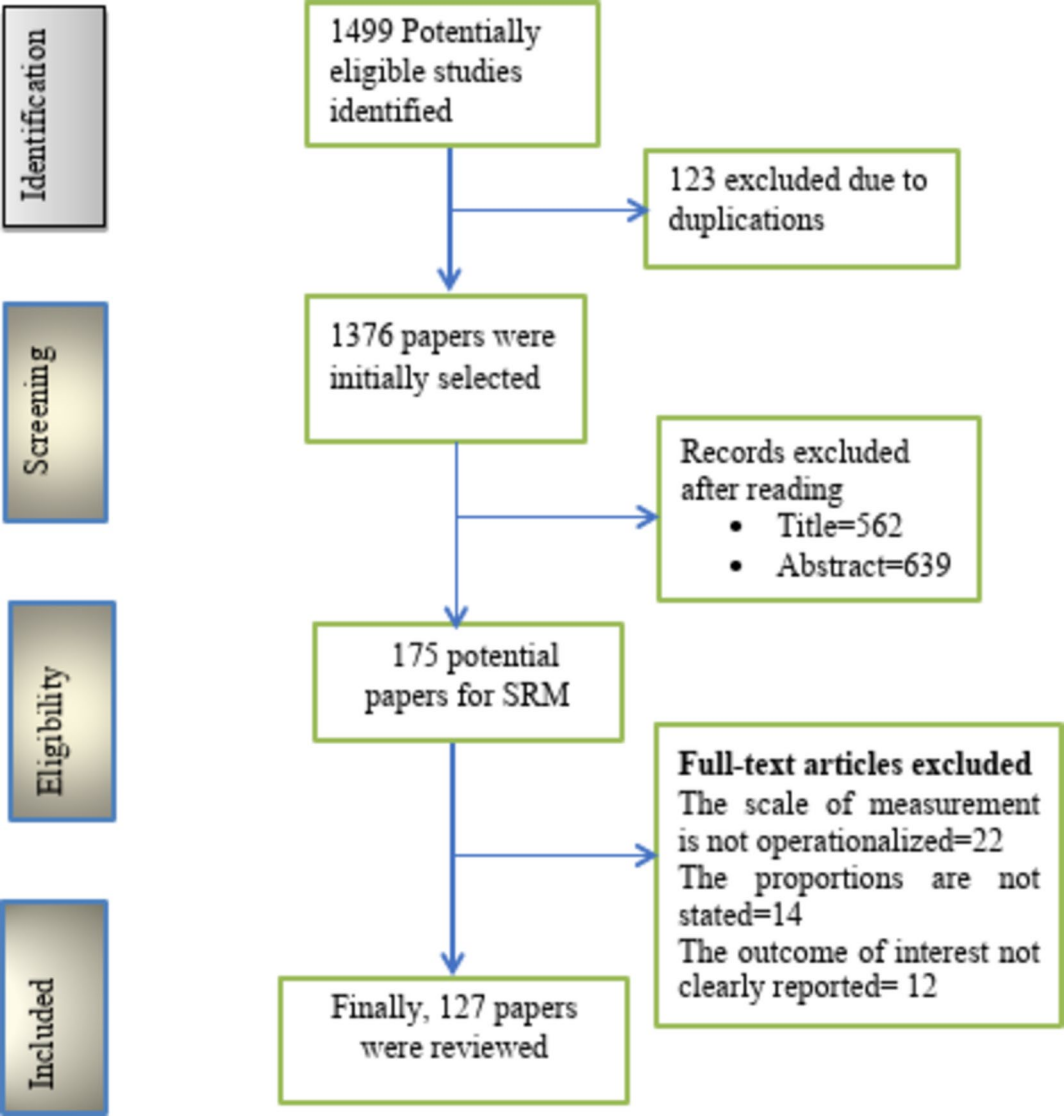
PRISMA flow diagram to illustrate the study selection process

This systematic review and meta-analysis were performed to determine the pooled prevalence of Nocturnal enuresis and its associated factors among children and adolescents in the world. The study was conducted based on the preferred reporting items for systematic reviews and meta-analyses (PRISMA) guidelines recommendation (Fig. 1). This systematic review and meta-analysis had been registered (CRD42024603498) on the International Prospective Register of Systematic Reviews (PROSPERO) protocol.

### Inclusion and exclusion criteria

This study includes all original research articles conducted elsewhere in the world that fulfilled the inclusion criteria. We included studies conducted among children and adolescents aged 5 to 18, published in English, used observational studies including longitudinal cohort, cross-sectional, and case-control studies, and were available in electronic resources regardless of the year of publication. In this systematic review and meta-analysis, articles that had no clearly stated outcome variable or no information on the practice outcome, low quality, and studies difficult to extract necessary information due to no full-text access were excluded.

### Search strategy and source of information

The search strategy was formulated using an adapted “PEOS” framework (population, exposure, outcomes, study design, and setting) to create the MeSH terms needed to identify relevant studies in the database, as outlined below:

*Population*: Children and adolescents.

*Exposure*: Nocturnal enuresis, bedwetting, sleeping disorders, nighttime urinary incontinence.

*Outcome*: Prevalence, incidence, epidemiology; associated factors, predictors, barriers, or determinants.

*Study design*: Observational studies.

*Setting (context)*: Worldwide.

To identify relevant primary studies, we developed the following review questions based on the above format:

What is the global prevalence of nocturnal enuresis among children and adolescents?

What associated factors contribute to nocturnal enuresis or bedwetting in children and adolescents?

Then, primary studies were searched using PubMed Central, Cochrane Library, Web of Science, Scopus, and Google Scholar searching databases. The search was conducted in electronic databases using the following free-text terms and Boolean operator strings: (“Nocturnal enuresis” OR “bedwetting” OR “sleeping disorders” OR “nighttime urinary incontinence”) AND (“children” OR “adolescents”) AND (“prevalence” OR “incidence” OR “epidemiology”) AND (“associated factors” OR “predictors” OR “barriers” OR “determinants”). After searching for accessible articles, all the retrieved articles were sorted, and the duplications were removed.

### Study selection and quality assessment

Three investigators, MAA, TEH, and YAD screened the studies by reviewing titles and abstracts to identify potentially relevant studies before full-text retrieval. They contacted the corresponding authors for clarification when further information was needed to assess eligibility. Any discrepancies between the investigators were resolved through thorough discussion to ensure consensus on inclusion or exclusion. Additionally, all retrieved articles were imported into EndNote version 20, where duplicate files were removed. The investigators then independently screened the articles based on their objectives and designs, reviewing titles, abstracts, and full texts to identify eligible studies according to predetermined inclusion criteria, after which the selected articles were compiled by both reviewers.

To ensure the quality of each study, we employed a modified Newcastle-Ottawa Scale (NOS) evaluated by three independent reviewers. Reviewers conducted a thorough assessmentof each article that met the inclusion criteria and aligned with the study objectives. Any discrepancies between the reviewers were addressed through discussion and adjudication of a third reviewer. The assessment criteria encompassed several key domains: representativeness of the sample, adequacy of sample size, response rates and characteristics of responders versus non-responders, the quality of measurement tools used for exposure or risk factor ascertainment, comparability of outcome groups based on study design, control of important confounding variables, outcome assessment, and the application of statistical tests. Each study was scored out of a total of 7 points, with a score exceeding 4 indicating a low risk of bias for inclusion. All studies included in the analysis demonstrated a low risk of bias, achieving scores of 4 to 7.

### Data extraction

The data extraction form was developed using a Microsoft Excel spreadsheet. Two independent reviewers (MAA and TEH) systematically extracted data from full-text articles. This process involved utilizing the data extraction form, which encompassed essential variables such as the name of the first author, year of publication, country of study, study design, sample size, age of the participants, sample distribution by sex, assessment criteria, types of nocturnal enuresis (monosymptomatic vs. polysymptomatic, primary vs. secondary), as well as prevalence and the number of cases reported. For each primary outcome, we recorded the prevalence, while adjusted odds ratios for each associated factor (secondary outcome) were noted, accompanied by 95% confidence intervals. Any discrepancies between the data extractors were addressed through discussion and consensus with another author.

### Statistical analysis

After extracting the data, we performed a meta-analysis using STATA version 17. Prevalence estimates were calculated alongside their corresponding standard errors (SE), derived from the formulas p = r/n and SE = √ p (1 − p)/n​​, where p represents the proportion, r is the total number of children and adolescents with Nocturnal enuresis, and n is the sample size. The meta-analysis results are presented as the pooled prevalence of Nocturnal enuresis, accompanied by 95% confidence intervals. A significance threshold was set at p-values less than 0.05.

We also examined the factors associated with Nocturnal enuresis using STATA version 17. A random effects model was employed to assess significant heterogeneity among the studies. Heterogeneity was assessed using the I² index and Cochran’s Q test. The I² statistic, which ranges from 0 to 100%, indicates the degree of heterogeneity, with 0% signifying no heterogeneity and 100% indicating considerable heterogeneity. I² values below 25% suggest low heterogeneity, values between 25% and 50% indicate moderate heterogeneity, and values exceeding 75% reflect high heterogeneity.

To minimize random variations across studies, we conducted subgroup analyses to evaluate the prevalence of nocturnal enuresis among children and adolescents by year of publication, and continent. The impact of individual studies on the overall prevalence estimate was assessed through a sensitivity analysis. Finally, Publication bias was assessed using funnel plots for symmetry Egger’s test and non-parametric trim and fill tests. While both methods were employed, Egger’s test is generally more reliable for detecting publication bias, as it presents the actual effect sizes and their precision. In contrast, the funnel plot provides a more subjective view of potential asymmetry.

## Results

### Literature search

This systematic review and meta-analysis followed the PRISMA guidelines. The authors searched PubMed Central, Web of Science, Cochrane, and Google Scholar, identifying 1499 papers on Nocturnal enuresis among children and adolescents and its associated factors. After removing duplicates, 1376 papers were screened. Of these, 562 papers were excluded based on their titles, and 639 were discarded after abstract review. Ultimately, 128 studies met the inclusion criteria and were included in the final analysis.

### Study characteristics

The final analysis comprised 127 studies involving 445,242 individuals across 39 countries distributed over six continents. Specifically, the breakdown of studies by region is 76 studies were conducted in 16 Asian countries, 25 in 7 African countries, 20 in 11 European countries, 2 studies from 2 North American countries, and 2 studies were conducted in 1 South American country. Additionally, 2 studies were included from 2 Australian countries. Overall, 117 studies were cross-sectional, 7 were case-control, and 3 were cohort studies. Regarding the year of studies, 9 studies were conducted before 2000, 36 studies were conducted during the period from 2000 to 2009, 71 studies were conducted from 2010 to 2019, and 11 studies were conducted from 2020 to 2024. The median number of participants per study was 1133 (ranging from 100 to 130,000). Almost half (48.7%) of the participants were female. Among the 127 studies, 113 examined both types of nocturnal enuresis, 13 focused exclusively on the primary type, and 1 study specifically addressed the secondary type. One hundred nineteen studies have been conducted since the year 2000, while the remaining nine studies were completed before that year (Table 1).

**Table 1:** Descriptive summary of one hundred twenty-seven studies included in the meta-analysis of the global prevalence of nocturnal enuresis and associated factors among children and adolescents

Author(year)	Country	Design	Sample size	Age	Male	Female	Type (Primary/secondary)	Criteria	Prevalence	Quality assessment score
Abdulaziz Alamri et al. (2017) [16]	SA	CS	555	5–15	348	247	Both	ICD-10	24%	7/7
Abdul-Kareem M Ali (2009) [17]	Iraqi	CS	1000	6–12	488	512	Both	ICD-10	22%	7/7
Abdullah Alshahrani et al. (2017) [18]	SA	CS	352	5–12	221	131	Both	ICD-10	18.50%	6/7
Ahlam Ismail et al. (2012) [19]	Egypt	CS	9340	6–12	5239	5341	Primary	DSM-IV	10.13%	7/7
Ahmed Hamed et al. (2016) [20]	Egypt	CS	4652	6–12	2331	2321	Both	ICD-10	18%	7/7
Alaa H. Abed et al. (2009) [21]	Iraqi	CS	942	5–15	516	426	Both	ICD-10	24%	7/7
Ali Gunes et al. (2009) [22]	Turkey	CS	562	6–16	413	149	Both	ICD-10	14.90%	7/7
Mahmoud E. Abu Salem et al. (2016) [23]	Egypt	NCC	325	6–12	188	137	Both	DSM-IV	15.40%	6/7
Afshin Azhir et al. (2006) [24]	Iran	CS	3103	6–12	1524	1579	Both	DSM-IV	5.30%	7/7
Anfal Nayir H. Alanazi et al. (2022) [25]	SA	CS	420	6–18	187	233	Both	DSM-IV	24%	7/7
Ashok N. Solanki and Sarzoo G. Desai (2012) [26]	India	CS	1258	5–12	869	389	Both	DSM-IV	11.13%	7/7
Ashraf H. Mohammed et al. (2014) [27]	Egypt	CS	450	6–12			Both	ICD-10	15.70%	5/7
Emin Ozkaya et al. (2013) [28]	Turkey	CS	886	6–14	488	398	Both	DSM-IV	19.75%	7/7
Avinash De Sousa et al. (2007) [29]	India	CS	1473	6–10	1086	387	Both	DSM-IV	6.50%	7/7
Ayten Erdogan et al. (2007) [30]	Turkey	CS	356	5–7	174	182	Both	DSM-IV	12.60%	6/7
B Gu¨mu¨s et al. (1999) [31]	Turkey	CS	1703	7–11	832	871	Both	DSM-IV	13.74%	7/7
Bassem abu Merhi et al. (2014) [32]	Lebanon	CS	7270	5–18	4362	2908	Both	ICD-10	5.30%	7/7
BB Kalo and H Bella (1996) [33]	SA	CS	640	7–11	320	320	Both	ICD-10	15%	5/7
Bharat Choudhary et al. (2005) [34]	India	CS	1346	5–10	838	508	Both	DSM-V	12.70%	7/7
C.I. Esezobor et al. (2014) [35]	Nigeria	CS	928	7–17	444	484	Both	ICCS	24.40%	7/7
Yazici M cenk et al. (2012) [36]	Turkey	CS	9210	7–14			Both	DSM-IV	7.50%	6/7
Cuneyt Ozden et al. (2007) [37]	Turkey	CS	1,339	6–12	647	692	Both	ICD-10	17.50%	7/7
Dayanand P. Nakate et al. (2018) [38]	India	CS	1430	5–15	732	698	Both	DSM-IV	11.40%	7/7
Denise M. Mota et al. (2004) [39]	Brazil	CS	3,602	6–7	1872	1730	Both	DSM-IV	10.60%	7/7
Dipak N. Khadke et al. (2012) [40]	India	CS	413	5–10			Both	DSM-IV	10.91%	5/7
Elham Alhifthy et al. (2021) [41]	SA	CS	505	5–18	314	191	Both	DSM-V	48%	7/7
Emad M. Hammad et al. (2019) [42]	Egypt	CS	1148	5–12	532	612	Both	ICCS	17.80%	7/7
EMEL GÜR et al. (2002) [43]	Turkey	CS	1576	6–16	822	754	Primary	ICD-10	5.40%	7/7
Etuk, I. S et al. (2011) [44]	Nigeria	CS	2780	6–12	1,536	1,244	Both	ICCS	6.70%	7/7
Fatemeh -rkashvand et al. (2017) [45]	Iran	CS	1080	6–8	665	415	Both	DSM-IV	10.60%	7/7
Faten Younis et al. (2020) [46]	Egypt	CS	510	6–12	237	273	Both	DSM-IV	14.30%	7/7
Gaonkar Neha V. et al. (2018) [47]	Australia	CS	300	6–15	191	109	Both	ICCS	12.67%	7/7
Gulumser Dolgun et al. (2011) [48]	Turkey	CS	420	5–13			Both	DSM-IV	16.20%	6/7
H.N. Al-Naqeeb et al. (1989) [49]	SA	CS	1261	6–10			Both	DSM-IV	9.70%	7/7
Hamsa Shaker Abdul-Nabi e al (2013) [50]	Basra	CC	675	6–7	340	335	Both	DSM-IV	9.48%	7/7
Hasan Mohamed Aljefri et al. (2013) [51]	Yemen	CC	832	6–15	391	441	Both	DSM-IV	28.60%	7/7
Hasmet Sarici et al. (2013) [52]	Turkey	CS	1984	6–13	1031	953	Both	ICCS	9.52%	7/7
Hui-Lung Tai et al. (2006) [53]	Taiwan	CS	8496	6–12			Both	ICD-10	6.80%	6/7
Hui-Mei Huang et al. (2020) [54]	China	CS	6568	5–12	3409	3159	Both	ICCS	3.99%	7/7
Ipek Ozunan Akil et al. (2014) [55]	Turkey	CS	416	7–15	216	200	Both	ICCS	16.60%	7/7
Irene Mbinya Nzamu (2012) [56]	Kenya	CS	400	6–14	202	198	Both	DSM-IV	14.50%	7/7
J. M. Chinawa et al. (2014) [57]	Nigeria	CS	245	6–12	151	94	Both	DSM-IV	22.80%	7/7
J. Marleen Linde et al. (2018) [58]	Netherlands	CS	240	8–17			Both	ICCS	1.70%	7/7
Jae Min Chung et al. (2006) [59]	Korea	CS	16,516	5–13	8260	8260	Both	ICCS	6.42%	7/7
Jian Guo Wen et al. (2005) [60]	China	CS	10,088	5–18	5144	4944	Primary	DSM-IV	4.07%	7/7
Juliet Essen and Catherine Peckham (1986) [61]	UK	CS	12,232	5–11			Primary	ICCS	12%	6/7
K 0 Osungbade and F 0 Oshiname (2003) [62]	Nigeria	CS	664	6–12	356	308	Both	ICCS	17.60%	7/7
K.U. Özkan et al. (2004) [63]	Turkey	CS	3449	6–11	1632	1,817	Both	DSM-III	9.80%	7/7
Karim Eldin Mohamed Ali Salih et al. (2013) [64]	Sudan	CC	816	5–12	323	493	Both	DSM-IV	5.90%	7/7
Katayoun Bakhtiar et al. (2013) [65]	Iran	CS	710	5–10	355	355	Both	DSM-IV	8%	7/7
Katja Karniˇcnik et al. (2012) [66]	Slovenia	CS	1248	6–15			Primary	Not specified	12.40%	4/7
Khalida Anwer Yousef et al. (2010) [67]	Yemen	CS	656	6–18	316	340	Both	Not specified	17.20%	5/7
Kharifah Mohammad Sherah et al. (2019) [68]	SA	CS	505	5–12	251	254	Both	DSM-IV	76.40%	7/7
Kursat B. Carman et al. (2017) [69]	Turkey	CS	2589	6–12	1258	1331	Both	Not specified	20.80%	6/7
Lutf M. Al-Zubairi et al. (2018) [70]	Yemen	CS	2689	7–12	781	1245	Both	Not specified	11.20%	6/7
M. R. Jarvelin et al. (1986) [71]	Finland	CS	3206	7			Both	Not specified	6.40%	6/7
Mahboobeh Firouzkouhi Moghaddam et al. (2014) [72]	Iran	CS	1133	7–12	566	567	Both	DSM-IV	5.60%	7/7
Mahmoodzadeh Hashem et al. (2013) [73]	Iran	CS	958	7–11	453	465	Both	Not specified	18.70%	7/7
Sevim Savaser et al. (2017) [74]	Turkey	CS	2750	11–14	1366	1384	Both	DSM-V	1.45%	7/7
Maja Miskulin et al. (2010) [75]	Croatia	CS	3011	6–7			Both	ICCS	1.20%	6/7
Malik Tajuddin et al. (2010) [76]	Pakistan	CS	1236	5–15			Both	ICCS	13.10%	6/7
Margaret W. Fockema et al. (2012) [77]	SA	CS	3389	6–10			Both	ICCS	16.00%	6/7
Mariana Lima Por-carrero et al. (2011) [78]	Brazil	CS	100	5–17			Both	Not specified	5%	4/7
Mazhar Nazir Chatta et al. (2016) [79]	Pakistan	CS	1550	6–15	782	768	Both	ICCS	25%	7/7
Miao Shang Su et al. (2011) [80]	China	CS	6147	6–11	3115	3032	Both	ICD-10	4.60%	7/7
Michel N Aloni et al. (2012) [81]	DR Congo	CS	415	6–12	196	219	Both	ICCS	26.26%	7/7
Mitsuru Kajiwara et al. (2016) [82]	Japan	CS	202	13–15	99	103	Both	ICD-10	3.00%	5/7
Mohammad Alkot and Mohsen Deeb (2012) [83]	Egypt	CS	723	6–18	353	370	Both	ICD-10	14.67%	7/7
Mohammad R. Safarinejad (2017) [84]	Iran	CS	6889	5–18	3341	3548	Both	ICD- 10	4.80%	7/7
Mona Madbouly Shahin et al. (2017) [85]	SA	CS	652	5–12	286	366	Both	DSM-IV	22.70%	7/7
Muna Ahmed Awn et al. (2018) [86]	Bahrain	CS	438	5–12	235	202	Both	DSM-IV	10.75%	7/7
Muntather Sadiq Alhejji et al. (2020) [87]	SA	CS	321	5–16	173	148	Both	DSM-IV	11.20%	6/7
Murat Unalacak et al. (2004) [88]	Turkey	CS	1247	7–12	607	640	Both	ICD- 10	8.90%	7/7
N Semoli et al. (2009) [89]	Slovenia	CS	1311	5			Primary	ICCS	8.70%	6/7
N. Pashapour et al. (2008 (90)	Iran	CS	3500	7–12	1829	1671	Both	ICCS	7.70%	7/7
Necmettin Penbegü et al. (2012) [91]	Turkey	CS	4203	6–15	2192	2011	Both	ICCS	25.90%	7/7
Nega Tezera Assimamaw et al. (2024) [92]	Ethiopia	CS	730	5–14	433	297	Both	DSM-V	22.20%	7/7
Hansakunachai, Tippawan et al. (2005) [93]	Thailand	CS	2417	5–15			Both	Not specified	3.90%	5/7
Issa Hazza and Hussein Tarawneh (2005) [94]	Jordan	CS	680	6–8			Primary	Not specified	23.80%	5/7
Murad, Mohammed, et al. (2017) [95]	Iraq	CS	360		180	180	Both	Not specified	7.50%	4/7
J B Devlin (1991) [96]	Ireland	CS	1806				Both	Not specified	13%	4/7
NH Mbibu et al. (2005) [97]	Nigeria	CS	1416	5–14			Both	Not specified	14.90%	4/7
Nistha Shrestha et al. (2020) [98]	Nepal	CS	305	11–16	186	119	Both	Not specified	3.93%	7/7
Nuru Hassen Ibrahim et al. (2021) [99]	Ethiopia	CS	866	6–15			Both	DSM-IV	26.60%	6/7
Ornatcha Sirimongkolchaiyakul et al. (2023) [100]	Thailand	CS	3009	5–15			Primary	ICCS	9.70%	6/7
Ou Anyanwu et al. (2015) [101]	Nigeria	CS	216	6–18			Both	Not specified	37%	4/7
P. Chang et al. (2015) [102]	Taiwan	CS	1176	6–11	624	552	Primary	Not specified	8%	6/7
Pietro Ferrara et al. (2019) [103]	Italy	CS	130,000	5–14			Both	ICCS	7.20%	6/7
Premala Sureshkumar et al. (2009) [104]	Australia	CS	2856	5–10			Both	ICD-10	18.20%	6/7
Qing Wei Wang et al. (2007) [105]	China	CS	8696	7–18			Both	ICCS	5.57%	6/7
R J rona et al. (1997) [106]	UK	CS	14,674	5–11	7463	7211	Both	Not specified	9.80%	6/7
Ravi Gupta et al. (2016) [107]	India	CS	831	8–13	423	408	Both	Not specified	8.70%	6/7
Reda Goweda et al. (2020) [108]	Egypt	CS	363	5–16			Both	DSM-IV	63.90%	5/7
Safaa Mohammed El-Sayed Ahmed et al. (2022) [109]	Egypt	CS	454	6–12			Both	Not specified	8.80%	6/7
Richard J. Butler et al. (2005) [110]	England	Cohort	8151	7.5			Both	DSM-IV	2.60%	6/7
S Mattsson (1994) [111]	Sweden	CS	242	7–15			Both	Not specified	7.90%	5/7
S.D. Lee et al. (2000) [112]	Korea	CS	7012	7–12	3547	3465	Both	Not specified	9.40%	6/7
Saad S. Al-Zahrani (2014) [113]	SA	CS	2701	7–12	1501	1200	Both	ICCS	7.81%	7/7
Salva-re Arena and Mario Patricolo (2017) [114]	SA	Cohort	128	5–14			Primary	ICCS	30.40%	5/7
Sameena Shah et al. (2018) [115]	Pakistan	CS	429	5–16	243	186	Both	ICCS	43%	7/7
Seçil Özkan et al. (2010) [116]	Turkey	CS	14,060	5–12	7060	7000	Primary	DSM-IV	9.00%	7/7
D.M. Fergusson et al. (1990) [117]	New Zealand	Cohort	929	5–10			Secondary	DSM-III	7.90%	6/7
Sedat Aydin et al. (2008) [118]	Turkey	CS	1132	5–14	585	547	Both	Not specified	9.18%	6/7
Sema Uğuralp et al. (2003) [119]	Turkey	CS	1377	5–9	703	674	Both	ICCS	5.20%	7/7
Shatha Abdul-Rahman et al. (2008) [120]	Iraqi	CS	596	6–8	399	197	Both	DSM-IV	13.80%	7/7
Shitanshu Srivastava et al. (2012) [121]	India	CS	1212	6–12	418	794	Primary	ICCS	12.60%	7/7
Sinead Hanafin (1998) [122]	Ireland	CS	6206	4–14			Both	Not specified	10.73%	6/7
Srirangam Shreeram et al. (2008) [123]	USA	CS	1136	8–11	560	576	Both	DSM-IV	5.45%	7/7
Stephanie Gonzalez Mejias et al. (2008) [124]	Dominican Republic	CS	655	5–11	332	323	Both	Not specified	27.90%	6/7
Sunayna Pandey et al. (2020) [125]	India	CS	1904	5–12			Both	DSM-5	6.67%	6/7
Tine Caroc Warner et al. (2019) [126]	Denmark	CS	6803	5–15	3479	3324	Both	Not specified	10.28%	6/7
Tsang-Wee Cher et al. (2002) [127]	Taiwan	CS	7225	6–12	3649	3576	Both	Not specified	5.50%	6/7
Uju Ifeoma Nnubia et al. (2024) [128]	Nigeria	CS	806	9–12	560	246	Both	Not specified	15%	6/7
Xi Zheng Wang et al. (2019) [129]	China	CS	18,016	5–18			Both	Not specified	7.30%	5/7
Xianchen Liu et al. (2000) [130]	China	CS	3344	6–16	1795	1549	Both	DSM-IV	4.30%	7/7
Y Kameswari (2003) [131]	Malaysia	CS	2487	7–12	1082	1405	Both	ICD-10	8%	7/7
Yusuf Cetin Doganer et al. (2015) [132]	Turkey	CS	2314	6–14	1123	1191	Both	Not specified	9.90%	6/7
Birhane G Hiwot et al. (2016) [133]	Ethiopia	CS	1520	5–17	797	723	Primary	DSM-5	6.30%	7/7
M. Mohammadpour et al. (2012) [134]	Iran	CS	250	8.6 ± 1.05			Both	Not specified	6.80%	5/7
Emam Ghoraishy F. et al. (2004) [135]	Iran	CS	1000	6–11			Both	Not specified	16.50%	6/7
Hakim A. et al. (2015) [136]	Iran	CS	200	8.6 ± 1.8			Both	Not specified	32%	6/7
Shafi Pour Z et al. (2014) [137]	Iran	CS	768	7–11			Both	Not specified	7.20%	5/7
Ranjbar kochaksaraei F et al. (2003) [138]	Iran	CS	1092	5–16			Both	Not specified	1.80%	5/7
Hashem M et al. (2013) [139]	Iran	CS	918	7–11			Both	Not specified	18.70%	5/7
Majeed Hameed and Bilal Mohammed (2019) [138]	Iraqi	CS	490	6–10	245	245	Both	DSM-IV	14.90%	7/7
Chizoma I. Eneh et al. (2015) [139]	Nigeria	CC	140	5–11			Both	DSM-IV	20.70%	6/7
Alaa A Salih (2011) [140]	Iraq	CS	610	6–12			Both	DSM-IV	20.80%	6/7

SA: Saudi Arabia UK: United Kingdom CS: Cross-sectional CC: case Control NCC Nisted Case-control ICCS: International Children’s Continence Society DSM: Diagnostic and Statistical Manual of Mental Disorders ICD: International Classification of Diseases

### Publication bias

**Fig. 2:**
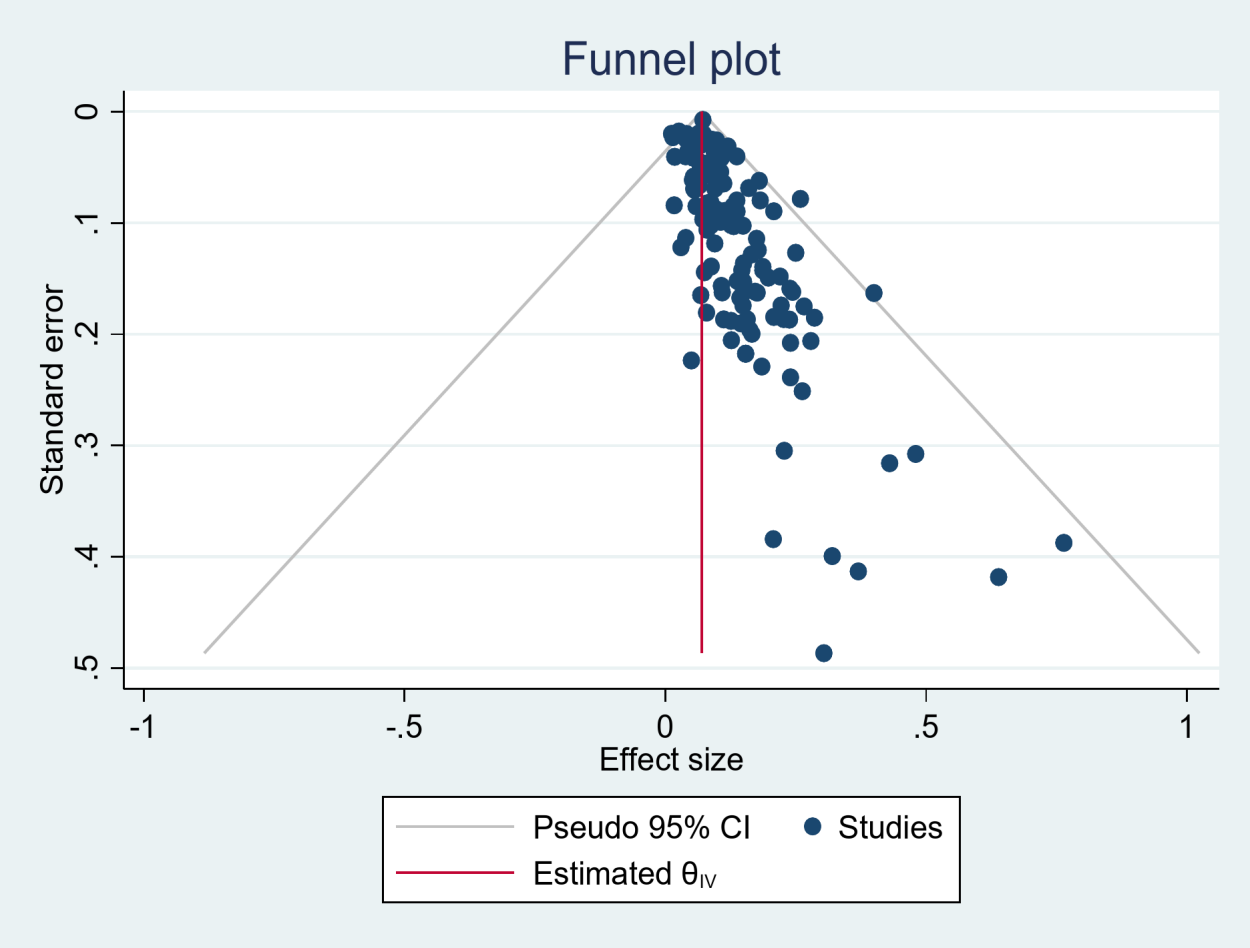
A funnel plot for publication bias for nocturnal enuresis among children and adolescents

The publication bias was evaluated using both funnel plot analysis and Egger’s regression test. The funnel plot revealed an asymmetric distribution, which intuitively suggests the presence of publication bias (Fig. 2). Additionally, Egger’s regression test yielded a p-value of 0.000, providing statistical evidence of publication bias. This combination of subjective and objective analyses underscores the likelihood of bias in the published literature.

To address the issue of potentially missing studies arising from publication bias indicated by the funnel plot and Egger’s test, we conducted a non-parametric trim and fill analysis to adjust the overall effect estimate. The analysis of publication bias through the nonparametric trim-and-fill method identified a total of 179 studies, with 52 studies imputed due to potential bias. The observed effect size was 0.069 (95% CI: (0.061, 0.077)), while the adjusted effect size, after accounting for the imputed studies, was 0.064, which falls within the confidence interval of the observed effect size. This suggests that the imputed studies had little to no impact on the overall effect size.

### Pooled prevalence of nocturnal enuresis

**Fig. 3:**
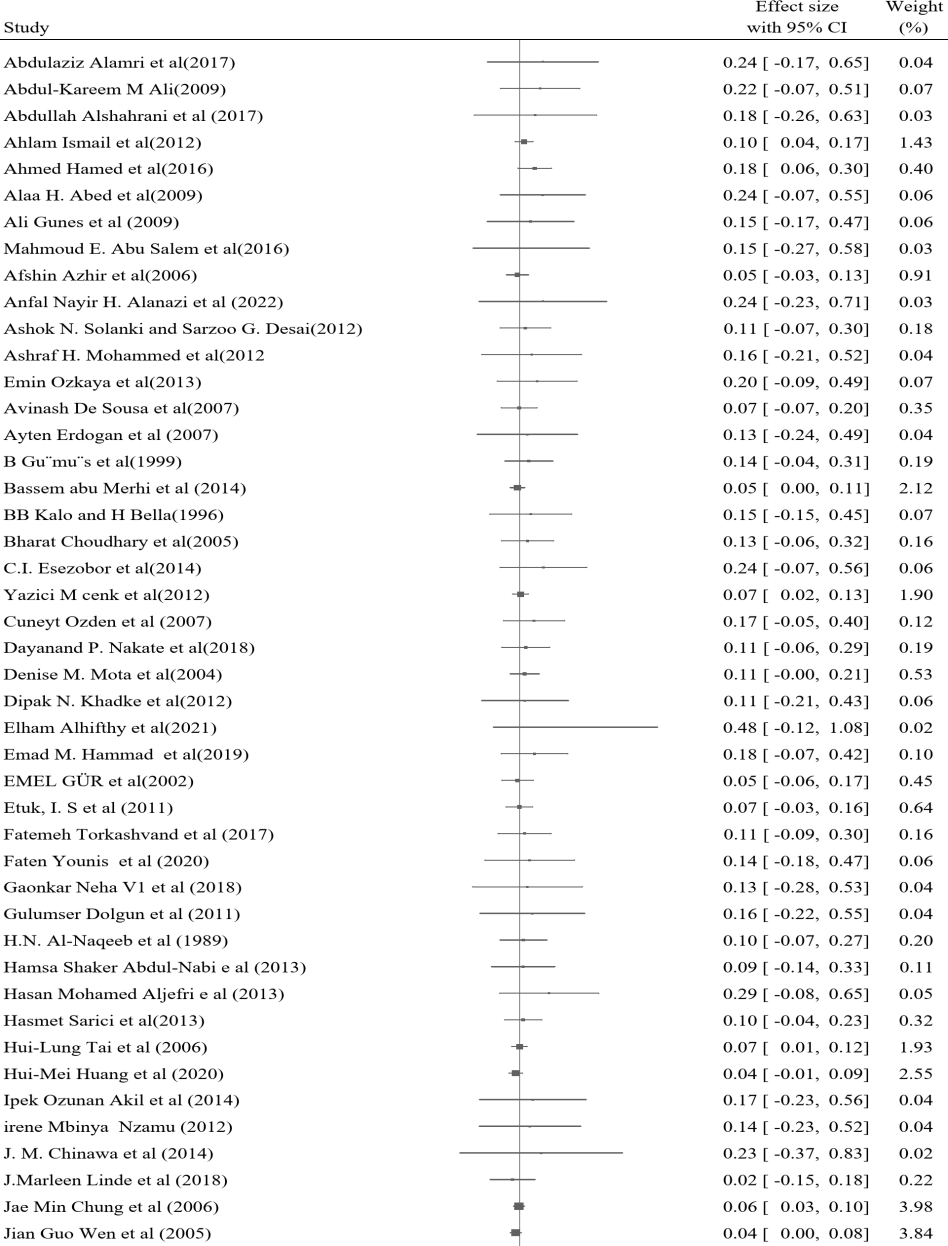

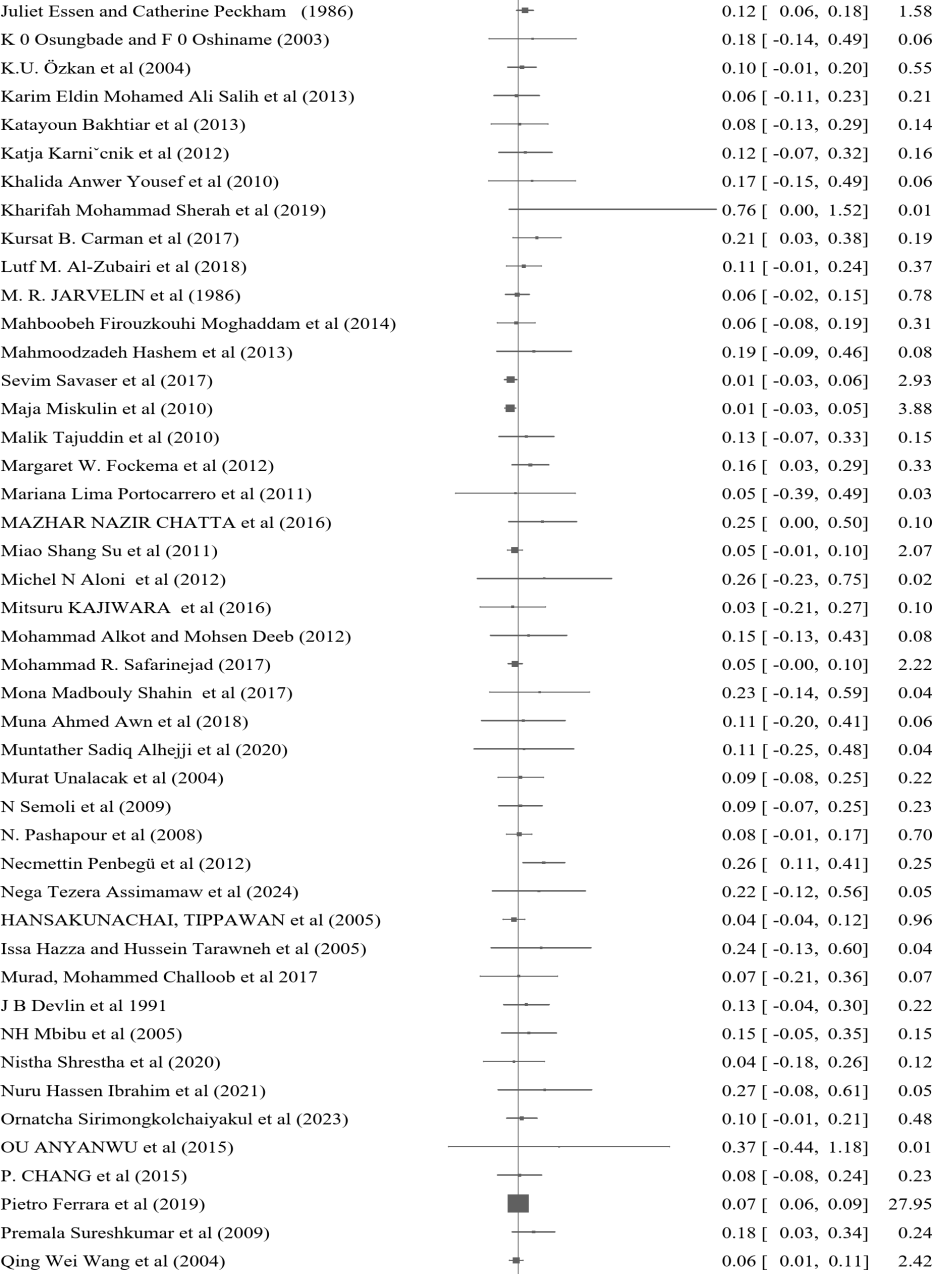

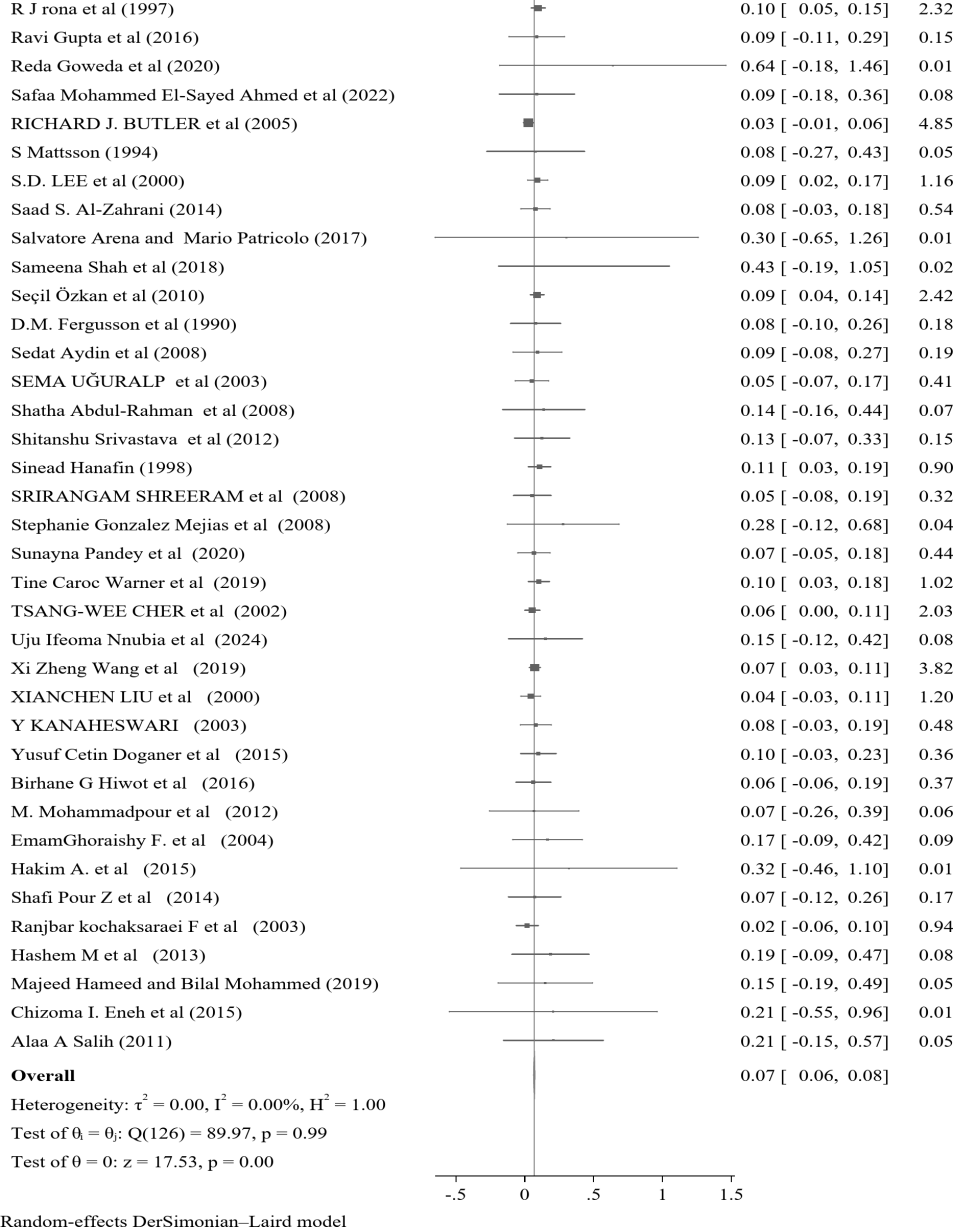
A forest plot for the pooled global prevalence of nocturnal enuresis among children and adolescents

The prevalence of Nocturnal enuresis among 127 individual studies ranged from 1.2–76.4%, with a pooled prevalence of 7.2% (95% CI: 6.2–8.1%), and there was no heterogeneity between the studies (*Q* = 90.12, *P* = 1.00, τ^2^ = 0.00, *I*^2^ = 0.00% and *H* = 1.00) (Fig. 3). Among the studies that specified both sex and the type of nocturnal enuresis, 60% of the cases were male, while 71.6% were classified as primary type.

### Subgroup analysis

**Fig. 4:**
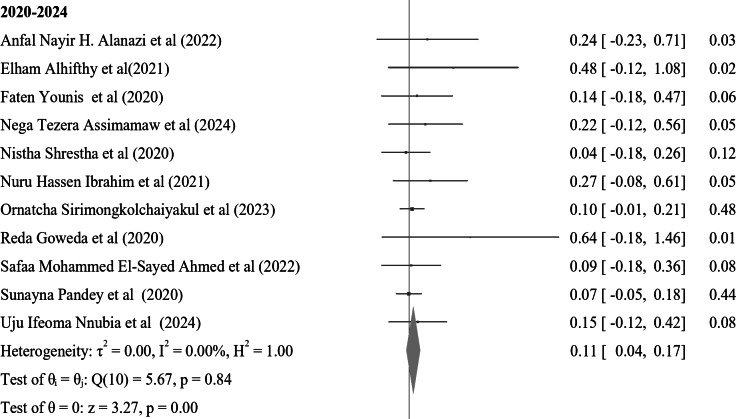

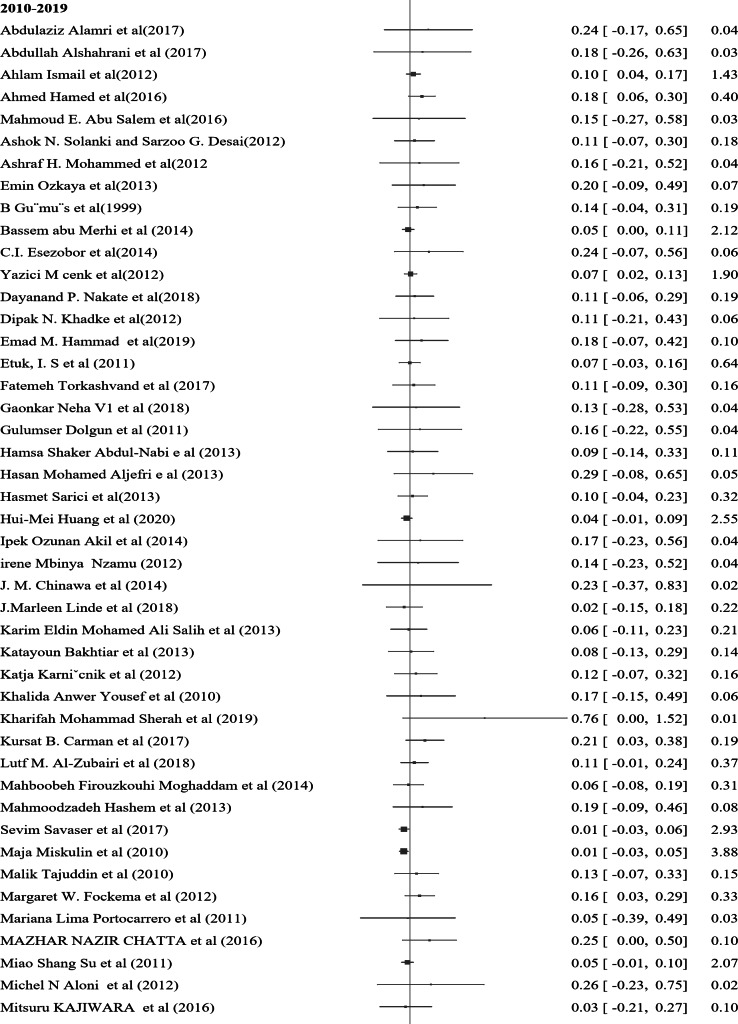

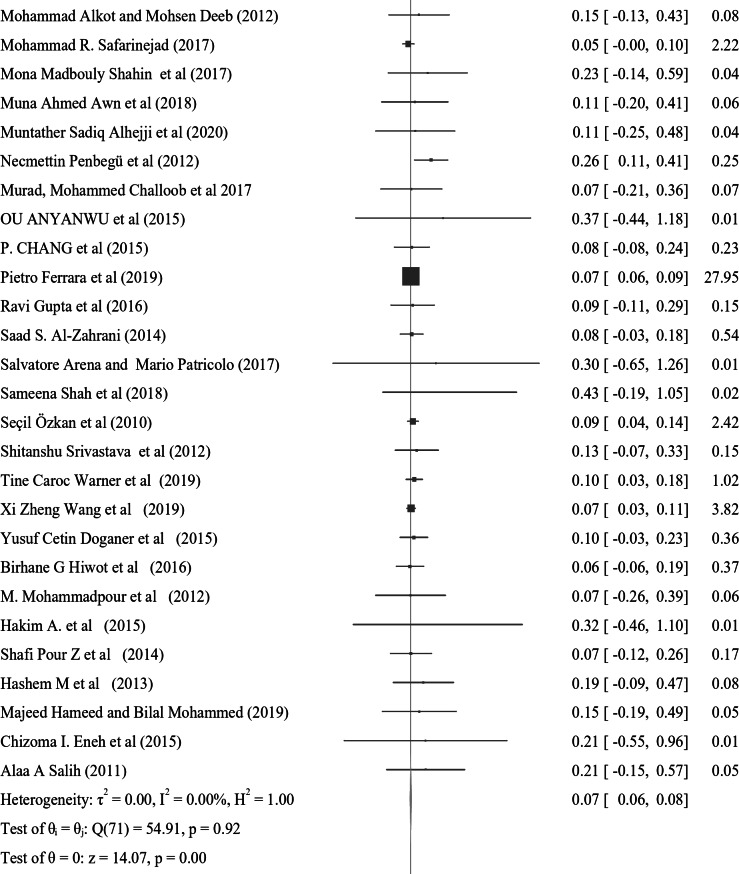

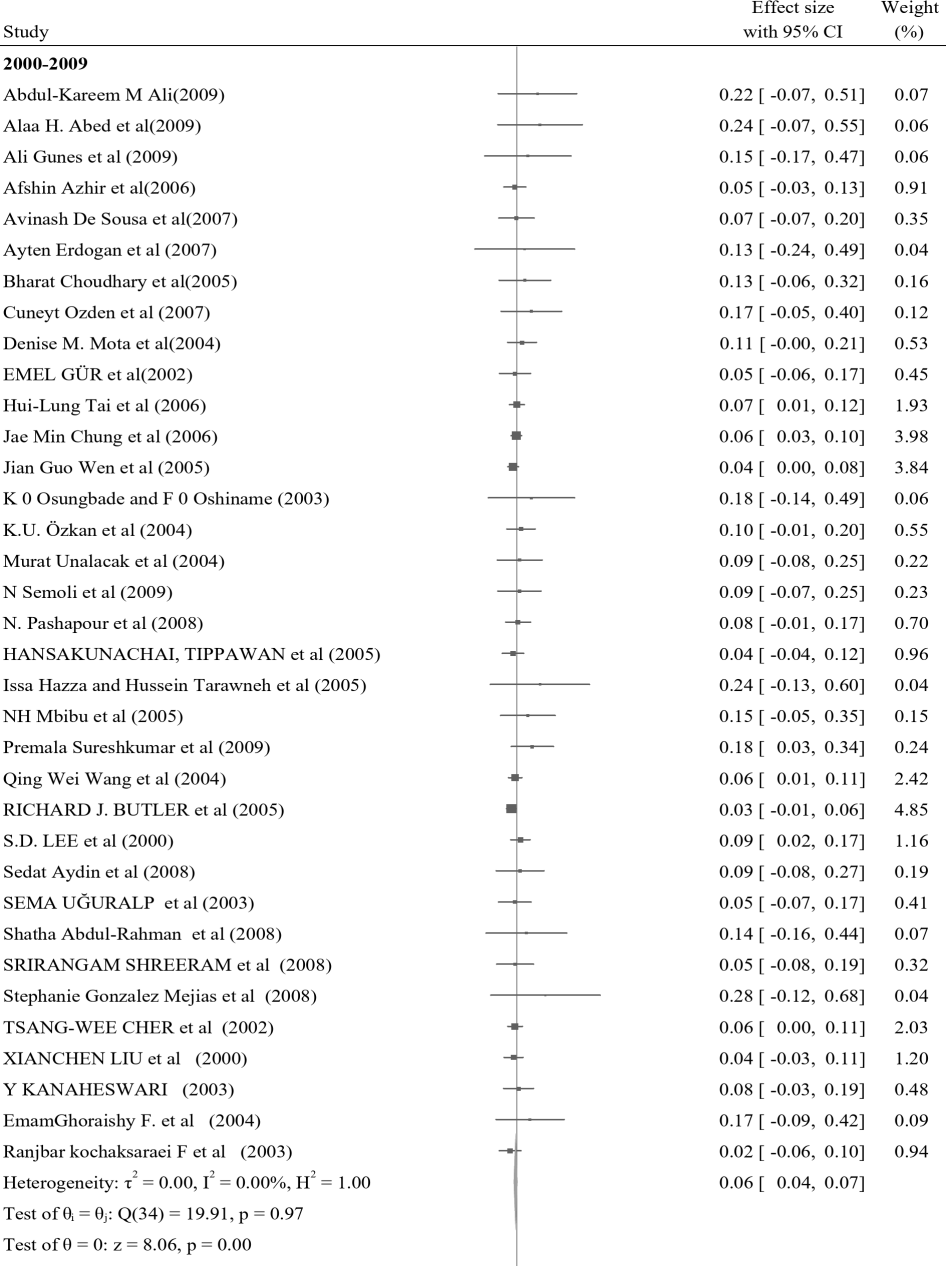

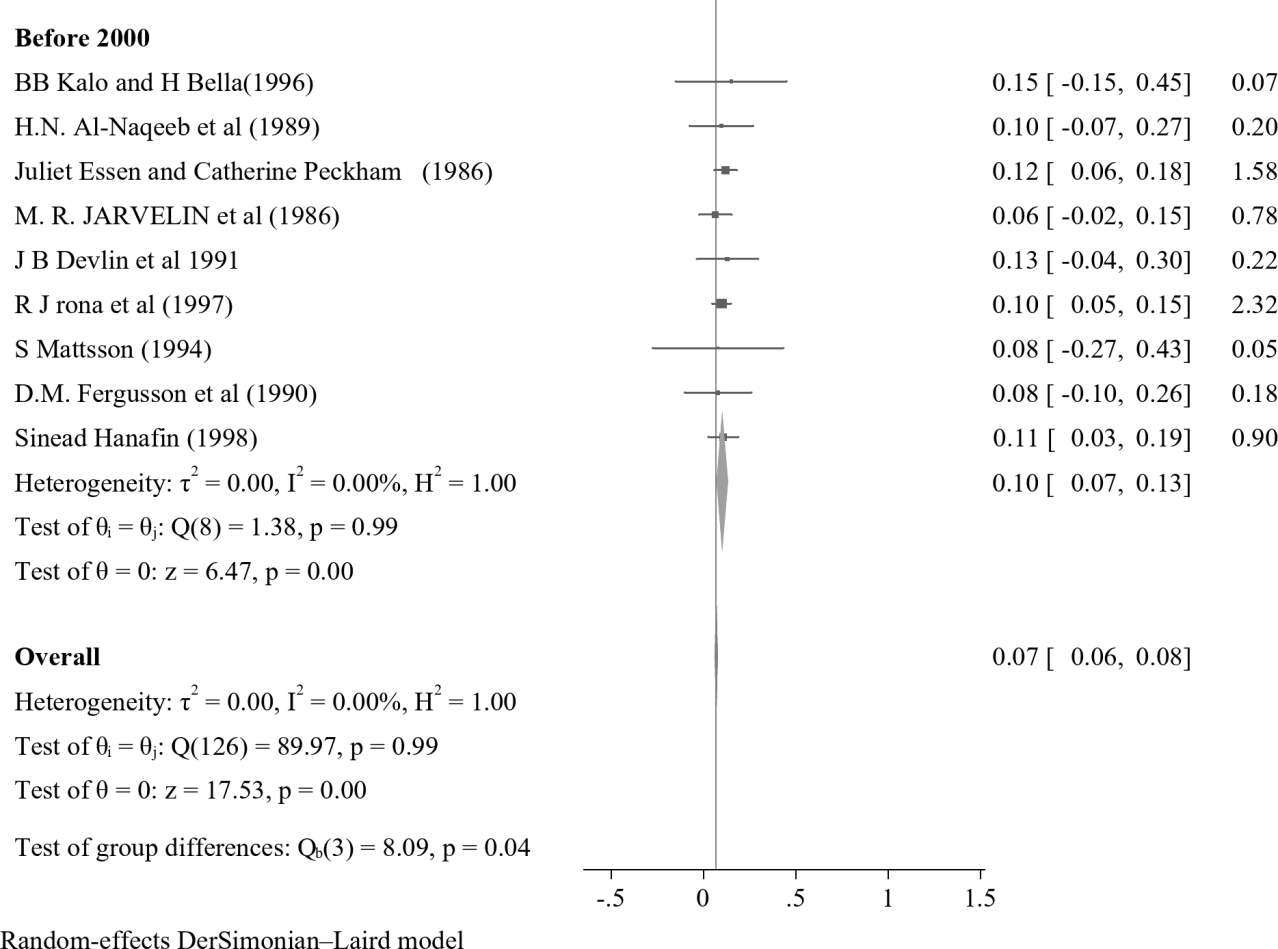
A forest plot for the sub-group analysis of the prevalence of nocturnal enuresis among children and adolescents based on the publication year of studies

**Fig. 5:**
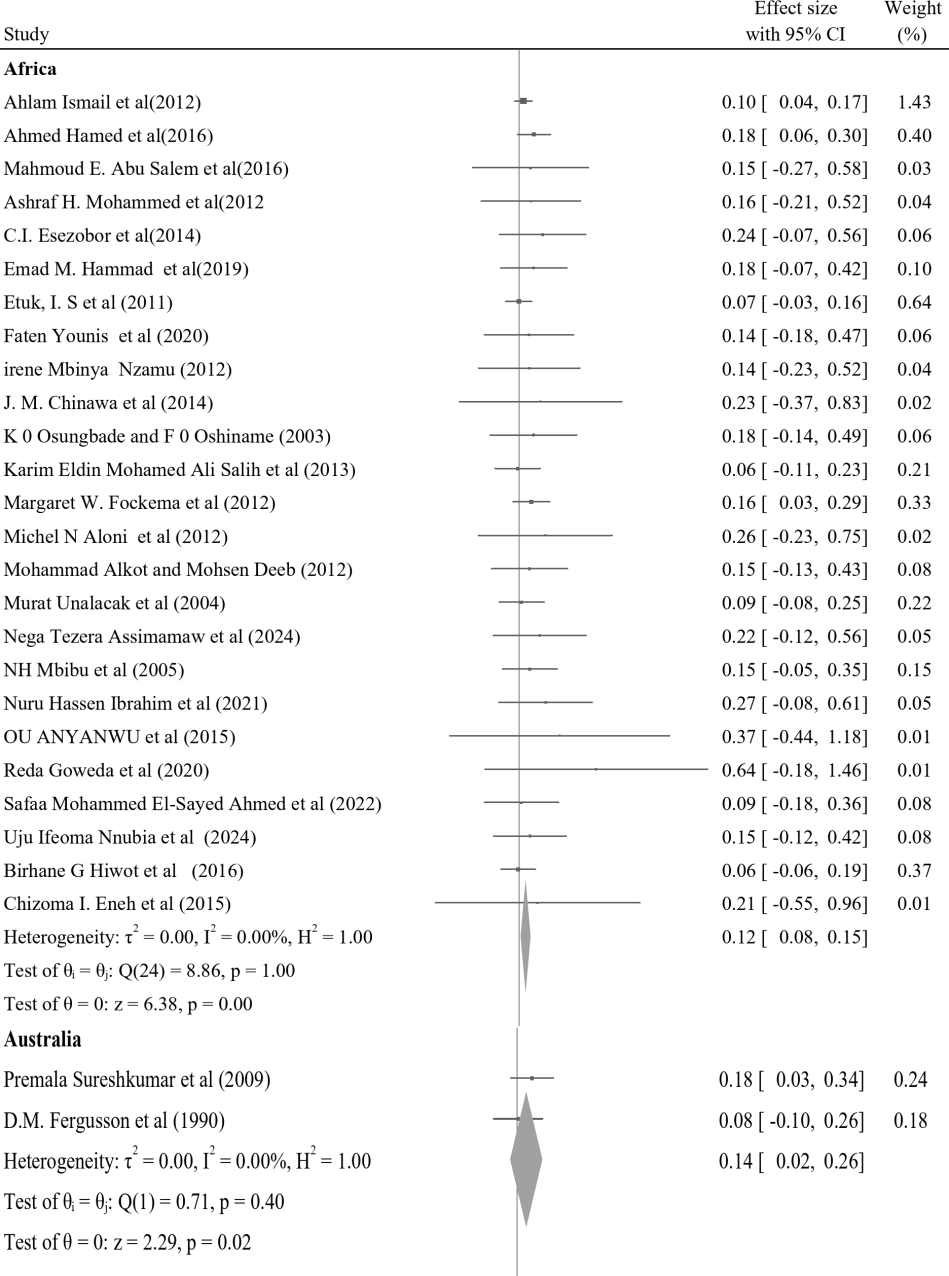

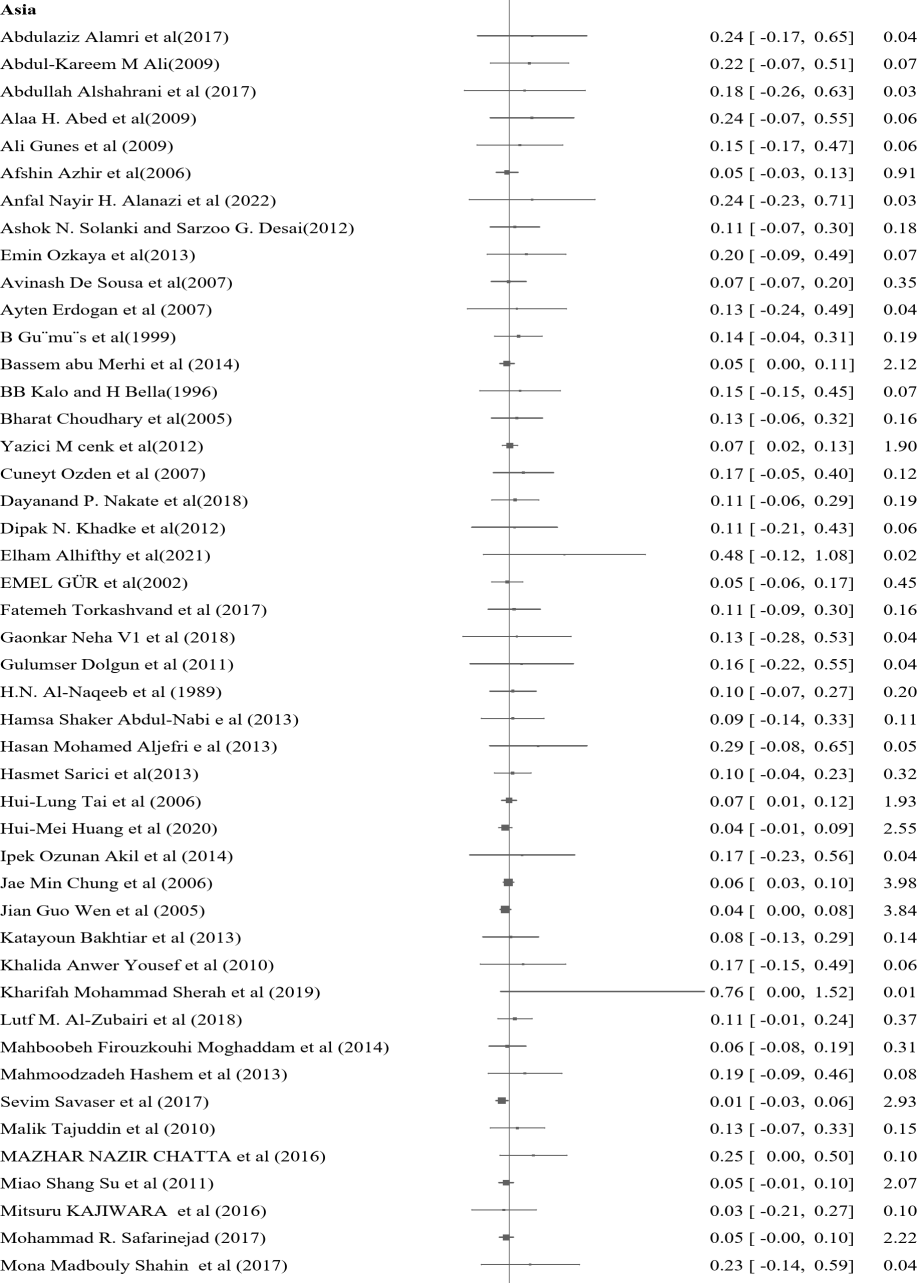

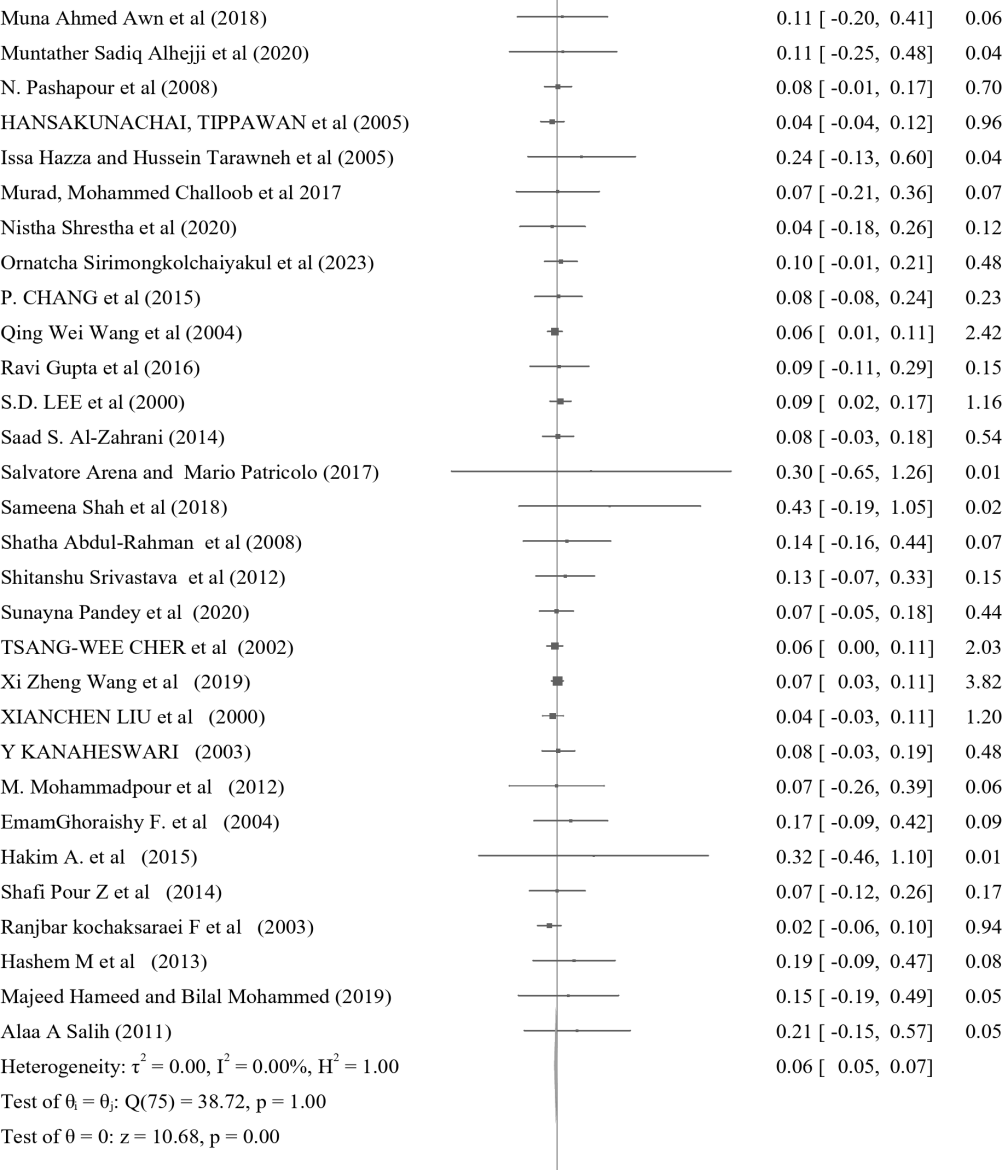

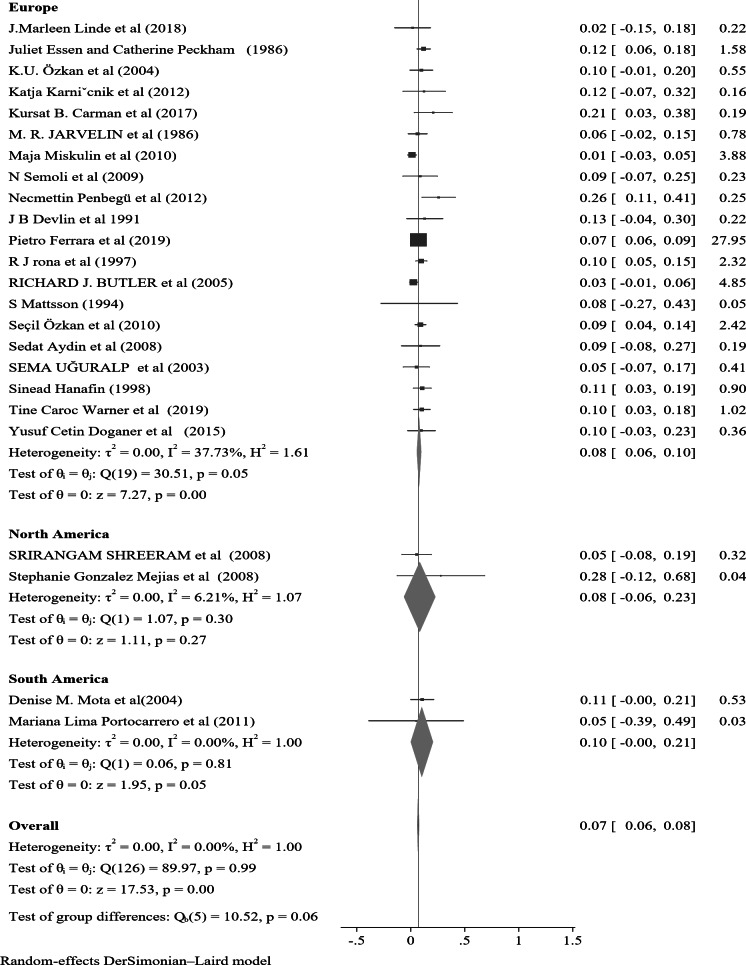
A forest plot for the sub-group analysis of the prevalence of nocturnal enuresis among children and adolescents based on continent

In the subgroup analysis of nocturnal enuresis (NE) using publication year, the pooled prevalence reported after 2019 indicates a higher rate of 0.11 (95% CI: 0.04–0.17, I² = 0.00%, *p* = 0.84). This contrasts with the pooled prevalence of 0.07 (95% CI: 0.06–0.08, I² = 0.00%, *p* = 0.93) observed in studies from 2010 to 2019. Furthermore, studies from 2000 to 2009 reveal a lower prevalence of 0.06 (95% CI: 0.04–0.07, I² = 0.00%, *p* = 0.98), while those conducted before 2000 report a prevalence of 0.10 (95% CI: 0.07–0.13, I² = 0.00%, *p* = 0.99) (Fig. 4). In a further subgroup analysis based on the continent, the pooled prevalence of nocturnal enuresis (NE) among African studies is notably higher at 0.12 (95% CI: 0.08–0.15, I² = 0.00%, *p* = 1.00). This contrasts sharply with the pooled prevalence of 0.06 (95% CI: 0.00-0.07, I² = 0.00%, *p* = 1.00) observed in studies from Asian countries. Additionally, studies from Europe report a prevalence of 0.08 (95% CI: 0.06–0.10, I² = 37.73%, *p* = 0.05), while studies from Australia indicate a higher prevalence of 0.14 (95% CI: 0.02–0.26, I² = 0.00%, *p* = 0.4). In North America, the prevalence stands at 0.08 (95% CI: 0.06–0.23, I² = 6.21%, *p* = 0.3), and studies from South America show a prevalence of 0.10 (95% CI: 0.00-0.21, I² = 0.00%, *p* = 1.00) (Fig. 5). These findings illustrate the considerable geographical variation in the prevalence of NE, with Africa exhibiting the highest rates among the continents analyzed.

### Sensitivity analysis

A sensitivity analysis was undertaken to assess the influence of outlying or potentially influential studies on the pooled prevalence estimate of nocturnal enuresis among children and adolescents. Employing a random effects model, the results of this analysis revealed that no influential studies were detected, as all point estimates remained within the boundaries of the 95% confidence interval. This finding indicates that the overall prevalence estimate is robust and not significantly impacted by any single study, thus reinforcing the reliability of the pooled results. Full results of the sensitivity analysis on the prevalence of NE among children and adolescents are provided in Fig. 6 of the supplementary file.

### Factors associated with nocturnal enuresis among children and adolescents

#### Family history of NE

**Fig. 6:**
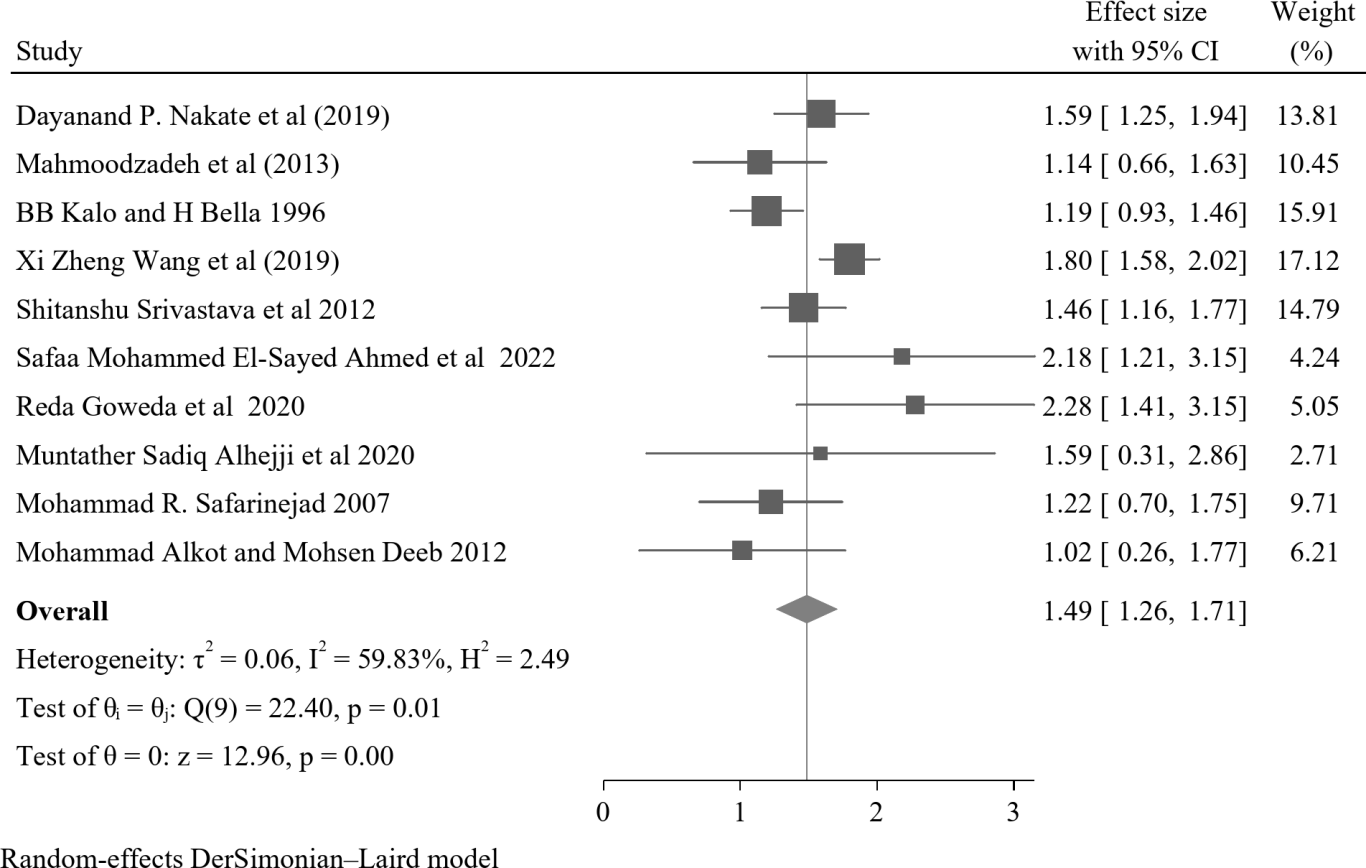
A forest plot for the association of family history with nocturnal enuresis among children and adolescents

Ten studies found a significant association between a family history of nocturnal enuresis and childhood nocturnal enuresis. Of these, the lowest and highest risk factors for children and adolescents with nocturnal enuresis were AOR 2.76(95% CI: 1.3–5.85) Mohammad Alkot and Mohsen Deeb 2012 and AOR 9.77(95% CI: 4.1-23.26) Reda Goweda et al. 2020 respectively compared with those who had no family history of NE. The forest plot pooled result of these ten studies showed that the overall estimate of AOR was 1.49(95% CI: 1.26–1.71); I^2^ = 59.83 and *p* = 0.01. I^2^ and p showed moderate heterogeneity (Fig. 6).

Concerning publication bias, the funnel plot analysis demonstrated asymmetrical distribution (Supplementary Fig. 8). However, the results from Egger’s regression test and the Begg test produced p-values of 0.53 and 0.72 respectively, both of which indicate the absence of publication bias. The Galbraith plot further corroborated this assessment by revealing no outliers (Supplementary Fig. 9).

### Positive UTI history

**Fig. 7:**
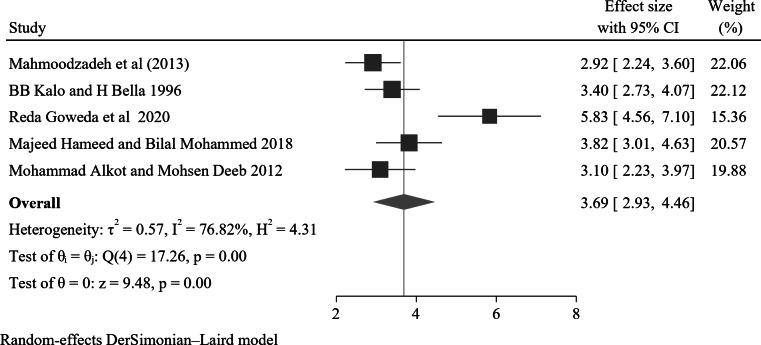
A forest plot for the association of positive UTI history with nocturnal enuresis

A total of five studies reported a significant association between positive urinary tract infection and NE. Of these, the lowest and highest risk factors for children and adolescents with nocturnal enuresis were AOR 2.92 (95% CI: 1.6–4.16) Mahmoodzadeh et al. (2013) and AOR 5.83 (95% CI: 1.52–22.33) Reda Goweda et al. 2020 respectively. The forest plot result of the studies showed that the overall effect size was 3.89, with a 95% CI (2.93–4.46). The heterogeneity measures, including I^2^ = 76.82% and P value = 0.00, suggest a high degree of variability in the effect sizes across the included studies (Fig. 7).

Regarding publication bias, the funnel plot analysis revealed a symmetrical distribution (Fig. 11 in the Supplementary file). Additionally, the results from Egger’s regression test and the Begg test results have p-values of 0.41 and 0.46 respectively, both of which indicate the absence of publication bias. The Galbraith plot corroborated this assessment by revealing no outliers (Fig. 12 in Supplementary file).

### Presence of stressful events

**Fig. 8:**
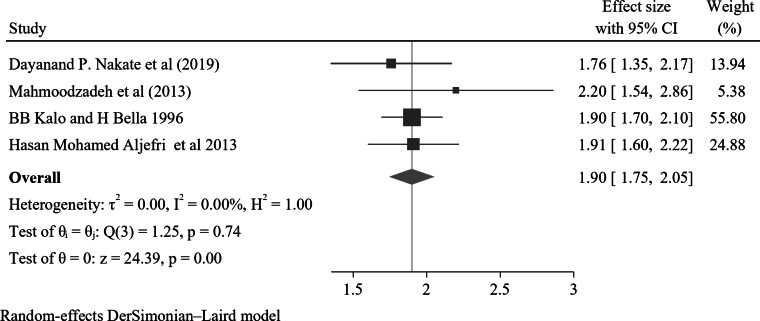
A forest plot for the association of parental death with nocturnal enuresis

The meta-analysis presented in the forest plot investigates the association between nocturnal enuresis with the presence of stressful events like the death of a loved one, parental divorce, relocation from a permanent home, and the presence of long-term illness. The overall pooled effect size of the studies was AOR = 1.90(95% CI; 1.75–2.05), I^2^ = 0.00%, and P value = 0.74, suggesting homogeneities in the effect sizes across the included studies (Fig. 8). The funnel plot analysis showed a symmetrical distribution (refer to Supplementary Fig. 14). Additionally, the results from Egger’s regression test resulted in p-values of 0.88, both of which indicate the absence of publication bias. The Galbraith plot also did not show any outliers (see Supplementary Fig. 15).

### Male sex

**Fig. 9:**
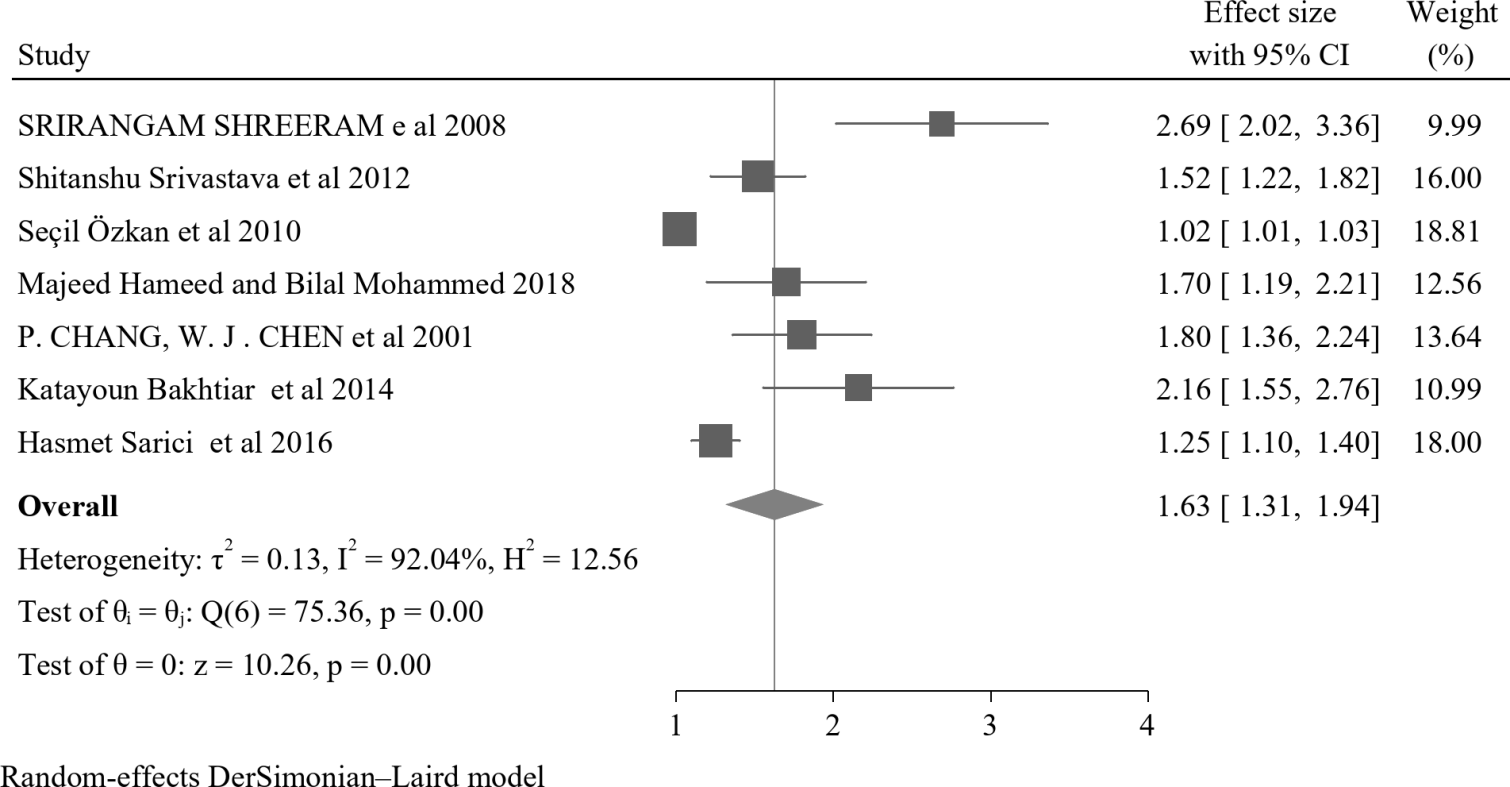
A forest plot for the association of male sex with nocturnal enuresis

A total of seven studies reported a significant association between male sex and NE. Of these, the lowest and highest risk factors for children and adolescents with nocturnal enuresis were AOR 1.02(95% CI: 1.01–1.03) Seçil Özkan et al. 2010 and AOR 2.69 (95% CI: 1.37–5.26) Srirangam Shreeram e al 2008 respectively. The forest plot result of the studies showed that the overall effect size was 1.63, with a 95% CI (1.31–1.94). The heterogeneity measures, including I^2^ = 92% and P value = 0.00, suggest a high degree of variability in the effect sizes across the included studies (Fig. 9). Concerning publication bias, the funnel plot analysis demonstrated asymmetrical distribution (Supplementary Fig. 17). Additionally, the results from Egger’s regression test and the Begg test produced p-values of 0.00 and 0.03 respectively, indicating publication bias. The Galbraith plot revealed no outliers (Supplementary Fig. 18).

### First birth order

**Fig. 10:**
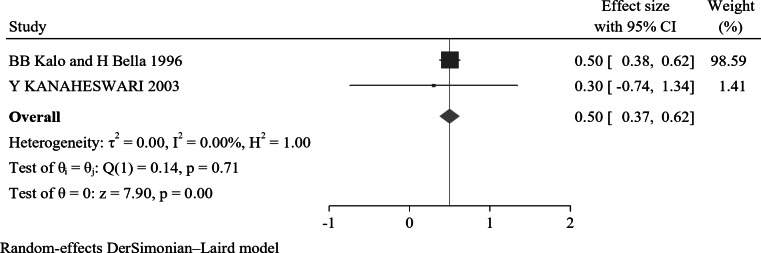
A forest plot for the association of first birth order with nocturnal enuresis

The forest plot result of the studies showed that the overall effect size was AOR 0.5 (95% CI: 0.37–0.62). The heterogeneity measures, including I^2^ = 0.00% and P value = 0.71, suggest a homogeneity in the effect sizes across the included studies (Fig. 10). Regarding publication bias, the funnel plot analysis revealed a symmetrical distribution (Supplementary Fig. 20). Additionally, the results from Egger’s regression test p-value of 0.83 indicate the absence of publication bias. The Galbraith plot corroborated this assessment by revealing no outliers (Supplementary Fig. 21).

## Discussion

Nocturnal enuresis, or bedwetting, is a significant global public health concern that remains under-recognized, particularly among children and adolescents. Despite its prevalence, the associated discrimination and stigma deter many young individuals from seeking help. Studies indicate that the rates of nocturnal enuresis are higher than previously acknowledged, with many affected children choosing not todisclose their condition due to fear of ridicule or misunderstanding. This lack of recognition impacts their emotional well-being and obstructs effective treatment.

This systematic review and meta-analysis, which incorporated 127 studies involving 445,242 children and adolescents globally, demonstrated a pooled prevalence of nocturnal enuresis of 7.2% (95% CI: 6.2–8.1%). The analysis employed a random effects model, confirming the homogeneity among the included studies, as indicated by the Q test (*p* = 1) and an I² statistic of 0.00%. Notably, this result is slightly lower than that reported in a systematic review conducted in Iran, which found a prevalence of 10.2%. This discrepancy may be attributed to the larger sample size utilized in the present analysis.

In the subgroup analysis of nocturnal enuresis (NE), the pooled prevalence reported for studies conducted after 2019 indicates a higher rate of 0.11 (95% CI: 0.04–0.17, I² = 0.00%, *p* = 0.84). This finding contrasts with the pooled prevalence of 0.07 (95% CI: 0.06–0.08, I² = 0.00%, *p* = 0.93) observed in studies from 2010 to 2019. Furthermore, studies from 2000 to 2009 reveal a lower prevalence of 0.06 (95% CI: 0.04–0.07, I² = 0.00%, *p* = 0.98), while those conducted before 2000 report a prevalence of 0.10 (95% CI: 0.07–0.13, I² = 0.00%, *p* = 0.99).

In a further subgroup analysis based on the continent, the pooled prevalence of NE among African studies is notably higher at 0.12 (95% CI: 0.08–0.15, I² = 0.00%, *p* = 1.00). This sharply contrasts with the pooled prevalence of 0.06 (95% CI: 0.00–0.07, I² = 0.00%, *p* = 1.00) observed in studies from Asian countries. Additionally, studies from Europe report a prevalence of 0.08 (95% CI: 0.06–0.10, I² = 37.73%, *p* = 0.05), while studies from Australia indicate a higher prevalence of 0.14 (95% CI: 0.02–0.26, I² = 0.00%, *p* = 0.40). In North America, the prevalence stands at 0.08 (95% CI: 0.06–0.23, I² = 6.21%, *p* = 0.30), and studies from South America show a prevalence of 0.10 (95% CI: 0.00–0.21, I² = 0.00%, *p* = 1.00).

The increase in prevalence rates post-2019 and the observed geographical variation may be attributed to heightened awareness and improved diagnostic practices. Additionally, the psychological and social impacts of global events, particularly the COVID-19 pandemic, alongside geopolitical factors such as conflicts and wars, have exacerbated these challenges, especially for children in affected regions where instability and trauma can further increase the prevalence of NE. Therefore, understanding these contextual influences is crucial for developing targeted interventions and providing effective support for children and adolescents experiencing nocturnal enuresis in the current global landscape.

Regarding the associated factors of NE, the pooled odds ratio indicates that children and adolescents with a family history of NE are nearly 1.5 times more likely to develop the condition compared to their counterparts without such a history, with an AOR of 1.49 (95% CI: 1.26–1.71). This finding is consistent with existing literature, which suggests a strong genetic predisposition to NE. Previous studies have established that familial patterns of bedwetting are common, highlighting the importance of genetic and environmental influences in the etiology of NE [9, 141, 142]. Understanding these familial links is crucial for identifying at-risk populations and developing targeted intervention strategies.

Furthermore, the pooled odds ratio indicates that children and adolescents with a history of UTIs) are nearly four times more likely to develop NE compared to those without a history of such infections, with an AOR of 3.89 (95% CI: 2.93–4.46). This association underscores the significant impact of UTIs on the development of NE, as recurrent infections can lead to bladder dysfunction and increased irritability of the urinary tract [5]. Prior studies have suggested that the inflammatory response associated with UTIs may contribute to alterations in bladder capacity and function, thereby increasing the likelihood of enuresis in affected individuals [12]. These findings highlight the importance of monitoring urinary health in children, particularly those with a history of UTIs, to mitigate the risk of developing NE.

Additionally, the pooled odds ratio indicates that children and adolescents who have experienced stressful events are nearly twice as likely to develop NE compared to those without such a history, with an adjusted odds ratio (AOR) of 1.90 (95% CI: 1.75–2.05). This finding underscores the significant impact that stressful experiences can have on a child’s psychological and emotional well-being, which may manifest in various behavioral and developmental issues, including NE. Research has demonstrated that adverse childhood experiences—such as the death of a loved one, parental divorce, relocation from a permanent home, and the presence of long-term illness—can lead to increased stress and anxiety, both of which are recognized risk factors for the development of enuresis [9, 11]. Consequently, understanding the implications of familial disruptions is crucial for clinicians and caregivers in identifying at-risk populations and implementing effective interventions to support affected children.

Furthermore, the pooled odds ratio indicates that male children and adolescents are approximately 1.63 times more likely to develop NE compared to their female counterparts, with an AOR of 1.63 (95% CI: 1.31–1.94). This finding is potentially due to biological, psychological, and social factors. Boys are more likely to experience delays in bladder maturation and may exhibit different coping mechanisms in response to stress. Additionally, societal expectations regarding gender behavior may influence reporting and diagnosis, further contributing to the observed disparity [144]. Understanding these gender differences is essential for developing targeted interventions and providing appropriate support for children affected by NE.

Finally, the pooled odds ratio indicates that children and adolescents who are first-born are 50% less likely to develop NE compared to those who are subsequent births, with an AOR of 0.50 (95% CI: 0.37–0.62). This finding suggests that birth order may play a significant role in the prevalence of NE, potentially due to differences in parenting practices, sibling dynamics, and environmental factors. Research has shown that first-born children often receive more focused attention and resources from parents, which may enhance emotional and developmental support during early childhood [143]. Understanding the implications of birth order can be valuable for clinicians in identifying at-risk populations and developing tailored interventions for families dealing with NE.

This study represents the first systematic review of the global prevalence of nocturnal enuresis (NE) among children and adolescents. We conducted a meta-analysis to synthesize prevalence estimates without imposing restrictions on publication dates, thereby maximizing the inclusion of relevant studies. The results exhibited homogeneity across the included article, which enhances the generalizability of our findings. Additionally, we performed subgroup analyses based on the year of publication and geographic regions, facilitating an understanding of trends in NE prevalence over decades and highlighting the burden of the condition in different continents.

However, one limitation of this meta-analysis is the variability in cut-off points and assessment criteria employed by researchers, which may differ based on the study’s geographical context. Furthermore, some articles presented challenges in obtaining full-length information due to accessibility issues.

## Conclusion

In this systematic review, the summary estimate of nocturnal enuresis among children and adolescents was approximately 7.2%. Family history, urinary tract infection, stressful events, birth order, and sex were statistically significant factors.

Given these findings, it is crucial for healthcare providers to implement routine screening for nocturnal enuresis, especially for children presenting with known risk factors such as a family history of the condition or a history of urinary tract infections. Early identification and intervention can help mitigate the psychological and social impacts of nocturnal enuresis, which can be profound and long-lasting. Furthermore, the development of targeted interventions and support mechanisms is essential. This may include educational resources for families, behavioral therapies, and, when appropriate, pharmacological treatments. Recognizing the multifactorial nature of nocturnal enuresis will allow clinicians to tailor their approaches to individual patients effectively, thereby improving outcomes and quality of life for affected children and their families. We recommend that future research focus on developing specific intervention strategies tailored to at-risk populations and further investigating the long-term psychological effects of NE on affectedchildren.

## Electronic supplementary material

Below is the link to the electronic supplementary material.

Supplementary Material 1

## Data Availability

Data is provided within the manuscript or supplementary information files, and additional data will be available upon the request of the corresponding author.
