# Supplementary material for: Global prevalence of nocturnal enuresis and associated factors among children and adolescents: a systematic review and meta-analysis
Source: Child Adolesc Psychiatry Ment Health. 2025 Mar 20;19:23. doi: 10.1186/s13034-025-00880-x (PMC11927296; doi:10.1186/s13034-025-00880-x)
Supplement: Supplementary file 1 — Supplementary Material 1 [file 13034_2025_880_MOESM1_ESM.docx]

**Figure 6**: Leave-one-out sensitivity analysis for the included studies

Figure 8: Funnel plot test for the association of family history with Nocturnal enuresis among children and adolescents

Figure 9: Galbraith plot test for the association of family history with Nocturnal enuresis among children and adolescents

Figure 11: Funnel plot test for the association of positive UTI history with Nocturnal enuresis among children and adolescents

Figure 12: Galbraith plot test for the association of positive UTI history with Nocturnal enuresis among children and adolescents

Figure 14: Funnel plot test for the association of parental death with Nocturnal enuresis among children and adolescents

Figure 15: Galbraith plot test for the association of parental death with Nocturnal enuresis among children and adolescents

Figure 17: Funnel plot test for the association of male sex with Nocturnal enuresis among children and adolescents

Figure 18: Galbraith plot test for the association of positive UTI history with Nocturnal enuresis among children and adolescents

Figure 20: Funnel plot test for the association of male sex with Nocturnal enuresis among children and adolescents

Figure 21: Galbraith plot test for the association of positive UTI history with Nocturnal enuresis among children and adolescents
